# Existence of Tripled Fixed Points for a Class of Condensing Operators in Banach Spaces

**DOI:** 10.1155/2014/541862

**Published:** 2014-09-14

**Authors:** Vatan Karakaya, Nour El Houda Bouzara, Kadri Doğan, Yunus Atalan

**Affiliations:** ^1^Department of Mathematical Engineering, Faculty of Chemistry-Metallurgical, Yildiz Technical University, 34210 Istanbul, Turkey; ^2^Department of Mathematics, Faculty of Science and Letters, Yildiz Technical University, 34210 Istanbul, Turkey

## Abstract

We give some results concerning the existence of tripled fixed points for a class of condensing operators in Banach spaces. Further, as an application, we study the existence of solutions for a general system of nonlinear integral equations.

## 1. Introduction and Preliminaries

Measures of noncompactness are very useful tools in functional analysis, for instance, in metric fixed point theory and in the theory of operator equations in Banach spaces. The first measure of noncompactness, denoted by *μ*, was defined and studied by Kuratowski [1] in 1930. In 1955, Banaś and Goebel [2] used the function *μ* to prove his fixed point theorem. Darbo's fixed point theorem [2] is a very important generalization of Schauder's fixed point theorem [3] and several authors had used this concept for the resolution of nonlinear equations, some of whom are Aghajani et al. [4, 5], Banaś [6], Banaś and Rzepka [7], Mursaleen and Mohiuddine [8], and many others. Recently in [9], Aghajani et al. give a generalization of Darbo's fixed point theorem. Moreover, they present some results on the existence of coupled fixed points for class of condensing operators. In this paper, we generalize these results to obtain the existence of tripled fixed points for the same class of operators.

Throughout this paper, *X* is assumed to be a Banach space and BC(*ℛ* ^+^) is the space of all real functions defined, bounded and continuous on *ℛ* ^+^. The family of bounded subset, closure, and closed convex hull of *X* are denoted by *ℬ*_*X*_, $\bar{X}$, and Conv*X*, respectively.

**Definition 1 (see [10]).**

Let *X* be a Banach space and *ℬ*_*X*_ the family of bounded subset of *X*. A map

(1) $$\begin{array}{c} \mu :B_{X}⟶\left[0,\infty \right) \end{array}$$

is called measure of noncompactness defined on *X* if it satisfies the following.

1. *μ*(*A*) = 0⇔*A* is a precompact set.
2. *A* ⊂ *B*⇒*μ*(*A*) ⩽ *μ*(*B*).
3. $\mu \left(A\right)=\mu \left(\bar{A}\right)$, ∀*A* ∈ *ℬ*_*X*_.
4. *μ*(Conv*A*) = *μ*(*A*).
5. *μ*(*λA* + (1 − *λ*)*B*) ⩽ *λμ*(*A*) + (1 − *λ*)*μ*(*B*), for *λ* ∈ [0,1].
6. Let (*A*_*n*_) be a sequence of closed sets from *ℬ*_*X*_ such that *A*_*n*+1_⊆*A*_*n*_, (*n*⩾1), and lim⁡_*n*→*∞*_ *μ*(*A*_*n*_) = 0. Then, the intersection set *A*_*∞*_ = ⋂_*n*=1_^*∞*^ *A*_*n*_ is nonempty and *A*_*∞*_ is precompact.

**Theorem 2 (see [2]).**

Let *C* be a nonempty closed, bounded, and convex subset of *X*. If  *T* : *C* → *C* is a continuous mapping

(2) $$\begin{array}{c} \mu \left(TA\right)⩽k\mu \left(A\right),\;k\in \left[0,1\right), \end{array}$$

then *T* has a fixed point.

**Theorem 3 (see [9]).**

Let *C*  be a nonempty closed, bounded, and convex subset of *X* and *T* : *C* → *C* a continuous mapping such that for any subset *A* of *C*

(3) $$\begin{array}{c} \mu \left(TA\right)⩽\beta \left(\mu \left(A\right)\right)\mu \left(A\right), \end{array}$$

where *β* : *ℛ*_+_ → [0,1; that is, *β*(*t*_*n*_) → 1 implies *t*_*n*_ → 0. Then, *T* has at least one fixed point.

The following result is a corollary of the previous theorem.

**Corollary 4 (see [9]).**

Let *C*  be a nonempty closed, bounded, and convex subset of *X* and *T* : *C* → *C* a continuous mapping such that for any subset *A* of *C*

(4) $$\begin{array}{c} \mu \left(TA\right)⩽\varphi \left(\mu \left(A\right)\right), \end{array}$$

where *φ* : *ℛ*_+_ → *ℛ*_+_ is a nondecreasing and upper semicontinuous functions; that is, for every *t* > 0, *φ*(*t*) < *t*. Then, *T* has at least one fixed point.

**Definition 5 (see [11]).**

A coupled fixed point of a mapping *G* : *X* × *X* → *X* is an element (*x*, *y*) ∈ *X* × *X* such that *G*(*x*, *y*) = *x* and *G*(*y*, *x*) = *y*.

**Theorem 6 (see [12]).**

Let *μ*_1_, *μ*_2_,…, *μ*_*n*_ be measures of noncompactness in Banach spaces *E*_1_, *E*_2_,…, *E*_*n*_, (respectively).

Then, the function

(5) $$\begin{array}{c} \tilde{\mu }\left(X\right)=F\left(\mu_{1}\left(X_{1}\right),\mu_{2}\left(X_{2}\right),\ldots ,\mu_{n}\left(X_{n}\right)\right), \end{array}$$

defines a measure of noncompactness in *E*_1_ × *E*_2_ × ⋯×*E*_*n*_, where *X*_*i*_ is the natural projection of *X* on *E*_*i*_, for *i* = 1,2,…, *n*, and *F* is a convex function defined by

(6) $$\begin{array}{c} F:{\left[0,\infty \right)}^{n}⟶\left[0,\infty \right), \end{array}$$

such that

(7) Fx1,x2,…,xn=0⟺xi=0,for  i=1,2,…,n.

**Remark 7 .**

Aghajani and Sabzali [13] illustrated the previous theorem by the following example. Let the mapping *F* be as follows:

(8) Fx,y=max⁡x,y, or  Fx,y=x+y,for  any  x,y∈0,∞2.

They showed that

(9) $$\begin{array}{c} \tilde{\mu }\left(X\right)=\max\left(\mu_{1}\left(X_{1}\right),\mu_{2}\left(X_{2}\right)\right), \end{array}$$

or

(10) $$\begin{array}{c} \tilde{\mu }\left(X\right)=\mu_{1}\left(X_{1}\right)+\mu_{2}\left(X_{2}\right) \end{array}$$

defines a measure of noncompactness in the space *E*_1_ × *E*_2_, where, for *i* = 1,2, *μ*_*i*_ are measure of noncompactness in *E*_*i*_  and *X*_*i*_, *i* = 1,2 are the natural projections of *X* on *E*_*i*_.

**Theorem 8 (see [9]).**

Let *Ω* be a nonempty, bounded, closed, and convex subset of a Banach space *E* and let *φ* : *ℛ* ^+^ → *ℛ* ^+^. Assume that *φ* is a nondecreasing and upper semicontinuous function. Let *G* : *Ω* × *Ω* → *Ω* be a continuous operator satisfying

(11) μGX1×X2⩽φμX1+μX22,X1,X2∈Ω,

for any measure of noncompactness *μ*. Then, *G* has at least a coupled fixed point.

## 2. Main Results

**Definition 9 (see [14]).**

A tripled (*x*, *y*, *z*) of a mapping *G* : *X* × *X* × *X* → *X* is called a tripled fixed point if

(12) Gx,y,z=x,Gy,x,z=y,Gz,y,x=z.

**Remark 10 .**

We can notice that by taking

(13) Fx,y,z=max⁡x,y,z, for  any  x,y,z∈0,∞3,

or

(14) $$\begin{array}{c} F\left(x,y,z\right)=x+y+z,\;\text{for}\;\;\text{any}\;\;\left(x,y,z\right)\in {\left[0,\infty \right)}^{3} \end{array}$$

*F* satisfies the conditions of Theorem 6. Thus, for a measure of noncompactness *μ*_*i*_  (*i* = 1,2, 3), we have that

(15) $$\begin{array}{c} \tilde{\mu }\left(X\right)=\max\left(\mu_{1}\left(X_{1}\right),\mu_{2}\left(X_{2}\right),\mu_{3}\left(X_{3}\right)\right), \end{array}$$

or

(16) $$\begin{array}{c} \tilde{\mu }\left(X\right)=\mu_{1}\left(X_{1}\right)+\mu_{2}\left(X_{2}\right)+\mu_{3}\left(X_{3}\right) \end{array}$$

defines a measure of noncompactness in the space *E* × *E* × *E* where *X*_*i*_, *i* = 1,2, 3 are the natural projections of *X* on *E*_*i*_.

So, we obtain the following theorem.

**Theorem 11 .**

Let *Ω* be a nonempty, bounded, closed, and convex subset of a Banach space *E* and let *φ* : *ℛ* ^+^ → *ℛ* ^+^ be a nondecreasing and upper semicontinuous function such that *φ*(*t*) < *t* for all *t* > 0. Then, for any measure of noncompactness *μ*, the continuous operator *G* : *Ω* × *Ω* × *Ω* → *Ω* satifying

(17) μGX1×X2×X3⩽φμX1+μX2+μX33,X1,X2,X3∈Ω

has at least a tripled fixed point.

**Proof**

To prove this theorem, let us define the measure of noncompactness $\tilde{\mu }$ by

(18) $$\begin{array}{c} \tilde{\mu }\left(X\right)=\mu_{1}\left(X_{1}\right)+\mu_{2}\left(X_{2}\right)+\mu_{3}\left(X_{3}\right) \end{array}$$

and the mapping $\tilde{G}:\Omega \times \Omega \times \Omega \to \Omega$

(19) $$\begin{array}{c} \tilde{G}\left(x,y,z\right)=\left(G\left(x,y,z\right),G\left(y,x,z\right),G\left(z,y,x\right)\right). \end{array}$$

Since

(20) μ~G~X⩽μ~GX1×X2×X3×GX2×X1×X3m00×GX3×X2×X1=μGX1×X2×X3+μGX2×X1×X3   +μGX3×X2×X1⩽φμX1+μX2+μX33   +φμX1+μX2+μX33   +φμX1+μX2+μX33=3φμX1+μX2+μX33

and ${\tilde{\mu }}^{\prime }=\left(1/3\right)\tilde{\mu }$ is a measure of noncompactness, we get

(21) $$\begin{array}{c} {\tilde{\mu }}^{\prime }\left(\tilde{G}\left(X\right)\right)⩽\varphi \left({\tilde{\mu }}^{\prime }\left(X\right)\right). \end{array}$$

By Corollary 4, we obtain that *G* has at least a tripled fixed point.

## 3. Applications

We can see an application of Theorem 11 in the study of existence of solutions for systems of integral equations defined on the Banach space BC(*ℛ* ^+^) endowed with the norm

(22) $$\begin{array}{c} ||x||=\sup\left\{\left|x\left(t\right)\right|:t>0\right\}. \end{array}$$

The measure of noncompactness on BC(*ℛ* ^+^) for a positive fixed *t* on *ℬ*_BC(*ℛ*^+^)_ is defined as follows:

(23) $$\begin{array}{c} \mu \left(X\right)=\omega_{0}\left(X\right)+\lim\underset{t\to \infty }{\sup}\mathrm{diam}X\left(t\right), \end{array}$$

such that

(24) diam⁡Xt=sup⁡xt−yt:x,y∈X,where  Xt=xt:x∈X.

Before defining *ω*_0_(*X*), we need first to introduce the modulus of continuity.

Let *x* ∈ *X* and *ϵ* > 0;

(25) ωTx,ϵ=sup⁡xt−xs:t,s∈0,T,t−s⩽ϵ,for  T>0,

is the modulus of continuity of *x* on [0, *T*] and let

(26) ωTX,ϵ=sup⁡ωTx,ϵ:x∈X,ω0TX=lim⁡ϵ→0ωTX,ϵ,ω0X=lim⁡T→∞ω0TX.

Assume that

(i) *ξ*, *η*, *q* : *ℛ*_+_ → *ℛ*_+_ are continuous and *ξ*(*t*) → *∞* as *t* → *∞*;
(ii) the function *ψ* : *ℛ* → *ℛ* is continuous and there exist positive *δ*, *α* such that
  (27) $$\begin{array}{c} \left|\psi \left(t_{1}\right)-\psi \left(t_{2}\right)\right|⩽\delta {\left|t_{1}-t_{2}\right|}^{\alpha }, \end{array}$$
  for any *t*_1_, *t*_2_ ∈ *ℛ*_+_;
(iii) *f* : *ℛ*_+_ × *ℛ* × *ℛ* × *ℛ* × *ℛ* × *ℛ* → *ℛ* is continuous and there exists a nondecareasing continuous function Φ : *ℛ* → *ℛ* with Φ(0) = 0, so that
  (28) ft,x1,x2,x3,x4−ft,y1,y2,y3,y4 ⩽12φx1−y1+x2−y2+x3−y3    +Φx4−y4;
(iv) the function defined by |*f*(*t*, 0,0, 0,0)| is bounded on *ℛ*_+_; that is,
  (29) $$\begin{array}{c} M_{1}=\sup\left\{f\left(t,0,0,0,0\right):t\in R_{+}\right\}<\infty ; \end{array}$$
(v) *h* : *ℛ*_+_ × *ℛ*_+_ × *ℛ* × *ℛ* → *ℛ* is a continuous function and there exists a positive solution *r*_0_ of the inequality
  (30) $$\begin{array}{c} \frac{1}{3}\varphi \left(3r\right)+M_{1}+\Phi \left(\delta D\right)⩽r, \end{array}$$
  where *D* is positive constant defined by the equality
  (31) D=sup⁡∫0qtt,s,xηs,yηs,zηsds:mD=sup⁡∫0qtt,s,xηs,yηs,zηsdst,s∈R+,x,y,z∈BCR+,lim⁡ϵ→∞∫0qtht,s,xηs,yηs,zηsmnm−ht,s,uηs,vηs,wηsds=0,
  uniformly with respect to *x*, *y*, *z*, *u*, *v*, *w* ∈ BC(*ℛ*_+_).

**Theorem 12 .**

Suppose that (i)–(v) hold; then the system of integral equations

(32) xt=ft,xξt,yξt,zξt,∫0qtht,s,xηs,yηs,zηsdsm=mψ∫0qtht,s,xηs,yηs,zηsds,yt=ft,yξt,xξt,zξt,∫0qtht,s,yηs,xηs,zηsdsm=mψ∫0qtht,s,yηs,xηs,zηsds,zt=ft,zξt,yξt,xξt,∫0qtht,s,zηs,yηs,xηsdshm=mψ∫0qtht,s,zηs,yηs,xηsds

has at least one solution in the space *BC*(*ℛ*_+_) × *BC*(*ℛ*_+_) × *BC*(*ℛ*_+_).

**Proof**

Let *G* : BC(*ℛ*_+_) × BC(*ℛ*_+_) × BC(*ℛ*_+_) → BC(*ℛ*_+_) be an operator defined by

(33) Gx,y,zt=ft,xξt,yξt,zξt,∫0qtht,s,xηs,yηs,zηsdsm=mψ∫0qtht,s,xηs,yηs,zηsds.

For (*x*, *y*, *z*) ∈ BC(*ℛ*_+_) × BC(*ℛ*_+_) × BC(*ℛ*_+_), let

(34) x,y,zBCR+×BCR+×BCR+=x∞+y∞+z∞.

We can easily prove that the solution of (32) in BC(*ℛ*_+_) × BC(*ℛ*_+_) × BC(*ℛ*_+_) is equivalent to the tripled fixed point of *G*.

Obviously, *G*(*x*, *y*, *z*)(*t*) is continuous for any (*x*, *y*, *z*) ∈ BC(*ℛ*_+_) × BC(*ℛ*_+_) × BC(*ℛ*_+_). Hence, we have

(35) Gx,y,zt⩽ft,xξt,yξt,zξt,∫0qtht,s,xηs,yηs,zηsdsm=mψ∫0qtht,s,xηs,yηs,zηsdsmmmmmm−∫0qtht,s,xηs,yηs,zηsdsft,0,0,0 +ft,0,0,0,0⩽12φxξt+yξt+yξt +Φψ∫0qtht,s,xηs,yηs,zηsds +ft,0,0,0,0⩽12φxξt+yξt+yξt +Φδ∫0qtht,s,xηs,yηs,zηsdsα +ft,0,0,0,0.

Then, by (29) and (30), we get

(36) Gx,y,z∞ ⩽13φx∞+y∞+z∞+M1+ΦδD⩽r0.

So, we obtain

(37) $$\begin{array}{c} G\left({\bar{B}}_{r_{0}}\times {\bar{B}}_{r_{0}}\times {\bar{B}}_{r_{0}}\right)\subset {\bar{B}}_{r_{0}}. \end{array}$$

Now, we prove that $G:{\bar{B}}_{r_{0}}\times {\bar{B}}_{r_{0}}\times {\bar{B}}_{r_{0}}\to {\bar{B}}_{r_{0}}$ is continuous. Let (*x*, *y*, *z*), $\left(u,v,w\right)\in {\bar{B}}_{r_{0}}\times {\bar{B}}_{r_{0}}\times {\bar{B}}_{r_{0}}$ such that, for *ɛ* > 0,

(38) $$\begin{array}{c} {||\left(x,y,z\right)-\left(u,v,w\right)||}_{{\bar{B}}_{r_{0}}\times {\bar{B}}_{r_{0}}\times {\bar{B}}_{r_{0}}}<\epsilon . \end{array}$$

Then,

(39) Gx,y,zt−Gu,v,wt=ft,xξt,yξt,zξt,∫0qtht,s,xηs,yηs,zηsdsm=mψ∫0qtht,s,xηs,yηs,zηsdsm=−ft,xξt,yξt,zξt,∫0qtht,s,xηs,yηs,zηsdsmmmmm=ψ∫0qtht,s,uηs,vηs,mmmmmmmmmm=∫0qtht,s,uηs,vηs,wηsds⩽12φxξt−uξt+yξt−vξtmmmm+xξt−uξtzξt−wξt +Φψ∫0qtht,s,xηs,yηs,zηsdsm=000mm−ψ∫0qtht,s,uηs,vηs,wηsds⩽12φxξt−uξt+yξt−vξtmmmm+xξt−uξtzξt−wξt +Φδ∫0qtht,s,xηs,yηs,zηsdsm=mmii−∫0qtht,s,uηs,vηs,wηsdsα.

Using condition (iii) and (29), there exists *T* > 0 such that if *t* > *T*, then

(40) Φδ∫0qtht,s,xηs,yηs,zηsdsα ⩽13ϵ,

for any *x*,*y*, *z* ∈ BC(*ℛ*_+_). We notice two cases.

*Case 1*. If *t* > *T*, then from (39) and (40)

(41) $$\begin{array}{c} \left|G\left(x,y,z\right)\left(t\right)-G\left(u,v,w\right)\left(t\right)\right|⩽\frac{1}{3}\varphi \left(\epsilon \right)+\frac{1}{3}\epsilon . \end{array}$$

*Case 2*. Similarly, for *t* ∈ [0, *T*], we have

(42) Gx,y,zt−Gu,v,wt⩽12φxξt−uξt+yξt−vξtmm+xξt−uξtzξt−wξt +Φδ∫0qtht,s,xηs,yηs,zηsdsmmmimm−∫0qtht,s,uηs,vηs,wηsdsα⩽12φϵ+ΦδqTβϵα<12ϵ+ΦδqTβϵα,

where *q*_*T*_ = sup⁡{*q*(*t*) : *t* ∈ [0, *T*]}, and

(43) βϵ=sup⁡ht,s,x,y,z−ht,s,u,v,w:mmmt∈0,T,s∈0,qT,mmmx,y,z,u,v,w∈−r0,r0,mmmx,y,z−u,v,w<ϵ.

Since *β* is continuous on [0, *T*] × [0, *q*_*T*_] × [−*r*_0_, *r*_0_] × [−*r*_0_, *r*_0_], we have *β*(*ϵ*) → 0 and *ϵ* → 0. Thus, using (iii), we get

(44) $$\begin{array}{c} \Phi \left(\delta {\left(q_{T}\beta \left(\epsilon \right)\right)}^{\alpha }\right)⟶0,\;\text{as}\;\;\epsilon ⟶0. \end{array}$$

Finally, from (42) and (41), we conclude that *G* is a continuous function from ${\bar{B}}_{r_{0}}\times {\bar{B}}_{r_{0}}\times {\bar{B}}_{r_{0}}$ into ${\bar{B}}_{r_{0}}$.

Now, we show that the map *G* satisfies all the conditions of Theorem 11. To do this, for an arbitrary fixed *T* > 0 and *ϵ* > 0, assume that *X*_1_, *X*_2_, and *X*_3_ are nonempty chosen subsets of ${\bar{B}}_{r_{0}}$ and *t*_1_, *t*_2_ ∈ [0, *T*], with |*t*_2_ − *t*_1_| ⩽ *ϵ*. Without loss of generality, let

(45) $$\begin{array}{c} q\left(t_{1}\right)<q\left(t_{2}\right). \end{array}$$

For an arbitrary (*x*, *y*, *z*) ∈ *X*_1_ × *X*_2_ × *X*_3_,

(46) Gx,y,zt1−Gx,y,zt2⩽ft1,xξt1,yξt1,zξt1,∫0qt1ht1,s,xηs,yηs,zηsdsm=mψ∫0qt1ht1,s,xηs,yηs,zηsdsmmi−ft1,xξt2,yξt2,zξt2,∫0qt1ht1,s,xηs,yηs,zηsdsmm=mmψ∫0qt1ht1,s,xηs,m=mmmmmimiiim∫0qt1ht1,s,xηs,yηs,zηsds +ft1,xξt2,yξt2,zξt2,∫0qt1ht1,s,xηs,yηs,zηsdsm=mmψ∫0qt1ht1,s,xηs,yηs,zηsdsmmmi−ft2,xξt2,yξt2,zξt2,∫0qt1ht1,s,xηs,yηs,zηsdsmmmmmm=ψ∫0qt1ht1,s,xηs,mmmmmmimmmm=m∫0qt1ht1,s,xηs,yηs,zηsds +ft2,xξt2,yξt2,zξt2,∫0qt1ht1,s,xηs,yηs,zηsdsm=mmψ∫0qt1ht1,s,xηs,yηs,zηsdsmmmi−ft2,xξt2,yξt2,zξt2,∫0qt1ht1,s,xηs,yηs,zηsdsmmmmmm=ψ∫0qt1ht1,s,xηs,mmmmmim=mmmmm∫0qt1ht1,s,xηs,yηs,zηsds +ft2,xξt2,yξt2,zξt2,∫0qt2ht2,s,xηs,yηs,zηsdsmmm=ψ∫0qt1ht1,s,xηs,yηs,zηsdsmmmi−ft2,xξt2,yξt2,zξt2,∫0qt2ht2,s,xηs,yηs,zηsdsmmmmm=mψ∫0qt2ht2,s,xηs,mmm=mimmmmmmm∫0qt1ht1,s,xηs,yηs,zηsds⩽13φxξt1−xξt2+yξt1−yξt2ωr0,D1Tmm=i+zξt1−zξt2+ωr0,D1Tf,ϵ +Φδ∫0qt2ht2,s,xηs,yηs,zηsmmmmmmmimm−ht1,s,xηs,yηs,mmmmmmmmmmi∫0qt2ht2,s,xηs,yηs,zηszηsdsα +Φδ∫qt1qt2ht1,s,xηs,yηs,zηsdsα⩽13φωTx,ωTξ,ϵ+ωTy,ωTξ,ϵ mmm+ωTz,ωTξ,ϵ+ωr0,D1Tf,ϵ +ΦδqTωr0Th,ϵα+ΦδUr0TωTq,ϵα,

where

(47) ωTξ,ϵ=sup⁡ξt2−ξt1:t1,t2⩽ϵ,t2−t1⩽ϵ,ωTx,ωTξ,ϵ=sup⁡xt2−xt1:t1,t2∈0,T,11mmixt2−xt111t2−t1⩽ωTξ,ϵ,D1=qTsup⁡ht,s,x,y,z,t∈0,T,s∈0,qT,mmmmx,y,z∈−r0,r0ωr0,D1Tf,ϵ=sup⁡ft2,x,y,z,d−ft1,x,y,z,d,mimit1,t2∈0,T,mmit2−t1⩽ϵ,x,y,z∈−r0,r0,mmid∈−D1,D1,ωr0Tf,ϵ=sup⁡ht2,s,x,y,z−ft1,s,x,y,z,mimit1,t2∈0,T,t2−t1⩽ϵ,mmis∈0,qT,x,y,z∈−r0,r0,Ur0T=sup⁡ht1,s,x,y,z:t1∈0,T,mmis∈0,qT,x,y,z∈−r0,r0,

we obtain

(48) ωTGX1×X2×X3,ϵ  ⩽13φωTX1,ωTξ,ϵ+ωTX2,ωTξ,ϵmmmmmm+ωTX3,ωTξ,ϵ+ωr0,D1Tf,ϵ    +ΦδqTωr0Th,ϵα+ΦδUr0TωTq,ϵα.    

Further, by the uniform continuity of *f* and *h* on the compact sets [0, *T*]  ×  [−*r*_0_, *r*_0_]  ×   [−*r*_0_, *r*_0_]  ×  [−*r*_0_, *r*_0_]  ×  [−*D*_1_, *D*_1_] and [0, *T*] × [0, *q*_*T*_] × [−*r*_0_, *r*_0_] × [−*r*_0_, *r*_0_] × [−*r*_0_, *r*_0_], respectively, we get *ω*_*r*_0_,*D*_1__^*T*^(*f*, *ϵ*) → 0 and *ω*_*r*_0__^*T*^(*h*, *ϵ*) → 0 as *ϵ* → 0.

Moreover, Φ is a nondecreasing continuous function with Φ(0) = 0 and (iii), and we obtain

(49) ΦδqTωr0Th,ϵα+ΦδUr0TωTq,ϵα⟶0,ϵ⟶0.

By (48), we get

(50) ω0TGX1×X2×X3 ⩽13φω0TX1+ω0TX2+ω0TX3.

Taking the limit *T* → *∞* in (50), we obtain

(51) ω0GX1×X2×X3 ⩽13φω0X1+ω0X2+ω0X3.

Then, for arbitrary (*x*, *y*, *z*), (*u*, *v*, *w*) ∈ *X*_1_ × *X*_2_ × *X*_3_, and *t* ∈ *ℛ*_+_, we have

(52) Gx,y,zt−Gu,v,wt⩽13φxξt−uξt+yξt−vξtmmmmm+zξt−wξt +Φδ∫0qtht,s,xηs,yηs,zηsmmmmmmmmm−ht,s,uηs,mmmmmmmmmmiiim∫0qtht,s,xηs,yηs,zηsvηs,wηsdsα⩽12φdiam⁡X1ξt+diam⁡X2ξt+diam⁡X3ξt +Φδ∫0qtht,s,xηs,yηs,zηsmmmmmmmmm−ht,s,uηs,mmmmmmmmmiiiiiim∫0qtht,s,xηs,yηs,zηsvηs,wηsdsα.

Since (*x*, *y*, *z*), (*u*, *v*, *w*), and *t* are arbitrary in (52),

(53) diam⁡GX1×X2×X3t⩽13φdiam⁡X1ξt+diam⁡X2ξt+diam⁡X3ξt +Φδ∫0qtht,s,xηs,yηs,zηsmmmmmmimmmm−ht,s,uηs,vηs,mmmmmmmm−−mmwηst,s,xηs,mmmmmmmm−−000000mmyηs,zηsds∫0qtht,s,xηs,yηs,zηsα.

Taking again *T* → *∞* in (53), we obtain

(54) lim⁡sup⁡⁡sup⁡t→∞diam⁡GX1×X2×X3t+ω0⩽13φlim⁡sup⁡t→∞diam⁡X1ξt+lim⁡sup⁡t→∞diam⁡X2ξtmmmmm+lim⁡sup⁡t→∞diam⁡X3ξt.

We conclude from (51) and (54) that

(55) lim⁡sup⁡t→∞ω0GX1×X2×X3t+ω0GX1×X2×X3⩽13φlim⁡sup⁡t→∞diam⁡X1ξt+lim⁡sup⁡t→∞diam⁡X2ξtmmmmm+lim⁡sup⁡t→∞diam⁡X3ξt +13φω0X1+ω0X2+ω0X3.

Since *φ* is a concave function, (55) implies

(56) lim⁡sup⁡t→∞diam⁡GX1×X2×X3t    +ω0GX1×X2×X3  ⩽φ13lim⁡sup⁡t→∞diam⁡X1ξt+ω0X1    +φ13lim⁡sup⁡t→∞diam⁡X2ξt+ω0X2    +φ13lim⁡sup⁡t→∞diam⁡X3ξt+ω0X3.

Finally, since *μ* is defined by

(57) $$\begin{array}{c} \mu \left(X\right)=\omega_{0}\left(X\right)+\lim\underset{t\to \infty }{\sup}\mathrm{diam}X\left(t\right), \end{array}$$

we get

(58) μGX1×X2×X3 ⩽φμX1+μX2+μX33.

Hence, by Theorem 11, *T* has at least a tripled fixed point in BC(*ℛ*_+_) × BC(*ℛ*_+_) × BC(*ℛ*_+_).

**Example 1 .**

We consider the following system of integral equations

(59) xt=13+t2xt+yt+zt+∫0Txsssinytcos⁡zt1+cos⁡2zsmmmm+es1+x2s1+sin2ysmmmm·1+cos⁡2zsmmm·et1+x2s1+sin2ysmmmmm·1+cos⁡2zs−1ds,yt=13+t2yt+xt+zt+∫0Tysssinxtcos⁡zt1+cos⁡2zsmmmm+es1+y2s1+sin2xsmmmm·1+cos⁡2zsmmm·et1+y2s1+sin2xsmmmmm·1+cos⁡2zs−1ds,zt=13+t2zt+yt+xt+∫0Tzsssinytcos⁡xt1+cos⁡2zsmmmm+es1+z2s1+sin2ysmmmm·1+cos⁡2xsmmm·et1+z2s1+sin2ysmmmmm·1+cos⁡2xs−1ds.

We notice that by taking

(60) ft,x,y,z,p=13+t2x+13y+13z+p,ht,s,x,y,z=xssinycos⁡z+es1+x21+sin2y1+cos⁡2zet1+x21+sin2y1+cos⁡2z,ηt=ξt=qt=Ψt=Φt=t,φt=t−3,

we get the system integral equations (32).

To solve this system, we need to verify conditions (i)–(v).

Obviously, *ξ*, *η*, *q* : *ℛ*_+_ → *ℛ*_+_ are continuous and *ξ* → *∞* as *t* → *∞*. Further, the function *ψ* : *ℛ* → *ℛ* is continuous for *δ* = *α* = 1, and we have

(61) $$\begin{array}{c} \left|\psi \left(t_{1}\right)-\psi \left(t_{2}\right)\right|⩽\delta {\left|t_{1}-t_{2}\right|}^{\alpha }, \end{array}$$

for any *t*_1_, *t*_2_ ∈ *ℛ*_+_. Conditions (i) and (ii) hold.

Now, let

(62) ft,x,y,z,p−ft,u,v,w,ρ=13+t2x+13y+13z+p−13+t2u+13v+13w+ρ⩽13x−u+y−v+z−w+p−ρ=13φx−u+y−v+z−w+Φp−ρ.

Then, (iii) also holds.

Moreover,

(63) $$\begin{array}{c} M_{1}=\sup\left|\left\{f\left(t,0,0,0,0\right):t\in R_{+}\right\}\right|=0; \end{array}$$

then, (iv) is valid.

Let us verify the last condition (v). First,

(64) ht,s,x,y,z−ht,s,u,v,w=xssinycos⁡z+es1+x21+sin2y1+cos⁡2zet1+x21+sin2y1+cos⁡2zmii−ussinvcos⁡w+es1+u21+sin2v1+cos⁡2wet1+u21+sin2v1+cos⁡2w=xssinycos⁡zet1+x21+sin2y1+cos⁡2zmii−ussinvcos⁡wet1+u21+sin2v1+cos⁡2w⩽x1+x2set−u1+u2set⩽12set+12set⩽set.

Hence,

(65) lim⁡t→∞∫0tht,s,xηs,yηs,zηslim⁡t→∞lim⁡t→∞−ht,s,uηs,vηs,wηsds ⩽lim⁡t→∞∫0tsetds=0.

Furthermore, for any *x*, *y*, *z* ∈ BC(*ℛ*_+_) × BC(*ℛ*_+_) × BC(*ℛ*_+_),

(66) ∫0tht,s,xηs,yηs,zηsds ⩽∫0tht,s,xηs,yηs,zηsds ⩽∫0ts2et+esetds=t24et+1−1et =t2+4et−44et.

Thus,

(67) sup⁡∫0tht,s,xηs,yηs,zηsds,mmiii∫0tht,s,xηs,yηs,zηsdst,s∈R+,x,y,z∈BCR+×BCR+×BCR+ =sup⁡t2+4et−44et,t∈R+=1.

It is easy to see that, for any *r* > 0, we have that

(68) $$\begin{array}{c} \frac{1}{3}\varphi \left(3r\right)+M_{1}+\Phi \left(\delta D\right)⩽r \end{array}$$

holds and condition (v) is valid.

Consequently, the system has at least one solution in BC(*ℛ*_+_) × BC(*ℛ*_+_) × BC(*ℛ*_+_).
